# Plumage and eggshell colouration covary with the level of sex-specific parental contributions to nest building in birds

**DOI:** 10.1007/s00114-024-01899-4

**Published:** 2024-02-27

**Authors:** Jenő Nagy, Mark E. Hauber, Viktor Löki, Mark C. Mainwaring

**Affiliations:** 1 HUN-REN-UD Conservation Biology Research Group, Department of Botany, University of Debrecen, Egyetem tér 1., H-4032 Debrecen, Hungary; 2 Advanced Science Research Center and Program in Psychology, Graduate Center of the City University of New York, New York, NY 10031 USA; 3 Wetland Ecology Research Group, HUN-REN Centre for Ecological Research, Institute of Aquatic Ecology, Bem tér 18/C, H-4026 Debrecen, Hungary; 4 School of Environmental and Natural Sciences, Bangor University, Bangor, LL57 2DG UK

**Keywords:** Birds, Colour dichromatism, Egg colour, Nest building, Parental care

## Abstract

Interspecific variation in sex-specific contributions to prenatal parental care, including avian nest building, is becoming increasingly better understood as we amass more information on more species. We examined whether sex-specific nest building contributions covary with the colouration of parents and their eggs in 521 species of Western Palearctic birds. Having colourful plumage and laying colourful eggs are costly because of the deposition of pigments in feathers and eggs and/or forming costly nanostructural substrates in feathers, and so it might be expected that those costs covary with the costs of nest building at the level of individuals and/or across species to produce of a suite of codivergent traits. Using a phylogenetically informed approach, we tested the hypothesis that species in which females alone invest energy building nests exhibit less sexual plumage dichromatism. However, we found comparative support for the opposite of this prediction. We then tested that species in which females alone build nests lay more colourful, and costlier, eggs because the dual costs of building nests and laying colourful eggs can only be borne by higher quality individuals. As expected, we found that species in which females build nests alone or together with males are more likely to lay colourfully pigmented eggs relative to species in which only males build nests. Finally, stochastic character mapping provided evidence of the repeated evolution of female-only nest building. Interspecific sex differences in plumage colouration therefore covary in a complex manner with female pre- (nest building) and post-copulatory (egg production) investment in reproduction.

## Introduction

Sex-specific contributions to the care of offspring vary dramatically both between and within parental taxa, and interspecific variation in the level of investment represents the evolutionary outcome of the benefits attained, and the costs accrued, from caring for offspring (Clutton-Brock 1991). Our understanding of interspecific variation in sex-specific contributions to care for vulnerable and dependent offspring is far better than of the building of dens, birthing chambers, or nest structures in which offspring are born or hatched and then reared (Guillette and Healy 2015). This is surprising because, for example, avian nest building is energetically costly (Mainwaring and Hartley 2013), and nest characteristics directly impact traits associated with offspring survival and thus also of parental fitness (Mainwaring et al. 2014; Guillette and Healy 2015). Males and females both contribute to provisioning offspring in most avian species (Clutton-Brock 1991; Ketterson and Nolan 1994; Fargevieille et al. 2023) and so both sexes may also be expected to contribute to nest building, particularly as physiological or anatomical specializations that differentially predispose one or the other of the sexes to build avian nests appear to be negligible (Clutton-Brock 1991).

Our understanding of interspecific variation in sex-specific contributions to avian nest building has dramatically increased in recent years (Soler et al. 1998, 2019; Lifjeld et al. 2019; Mainwaring et al. 2021). For example, Lifjeld et al. (2019) showed that males were less likely to contribute to nest building in species with higher levels of promiscuity. Meanwhile, species in which nest building is performed by females alone have shorter breeding seasons, higher breeding latitudes, large clutch sizes, and more female-only incubation (Mainwaring et al. 2021). In addition, female plumage colouration is associated with relative male nest building contributions, with male nest building occurring more often in species with colourful female plumage (Soler et al. 2019). Further, sex-specific nest building contributions influence the design of nests because species with biparental nest building contributions build more elaborate nests, possibly because sex-specific cognitive abilities result in nests built by two birds being more elaborate than nests built by one parent (Mainwaring et al. 2021). These studies have provided new insights into the sex-specific nest-building contributions.

Energetic costs may play a role in determining sex-specific contributions to nest building. This is because each of the sexes is expected to invest relatively less in the nest-building stage of reproduction, as part of a wider evolutionary aim to invest less in reproduction than their reproductive partner (Clutton-Brock 1991). When nest building is more complex and costly, alternatively, the sexes may come together, and both contribute to nest building when its production is complex and costly (Ketterson and Nolan 1994; Alonso-Alverez et al. 2004; Cuthill et al. 2017; Fargevieille et al. 2023). Depending on individual quality, across diverse life history traits, higher quality individuals may also invest more in specific components, to generate a suite of positively covarying costly traits. For example, species in which females contribute to nest building may also have more colourful plumage and accrue costs relative to species with duller female plumage. This is because there is larger energetic investment into greater pigmentation of feathers and/or because of the finer-scale arrangement of feather materials into nanostructures that adaptively scatter light to create bright structural colours and patterns (Fitzpatrick 1998; McGraw et al. 2005; Hill 2000; Fargevieille et al. 2023; Rincón-Rubio et al. 2023).

Plumage colouration, meanwhile, is a widely used way in which individuals signal their own quality either via melanin pigmented patterns (Fitzpatrick 1998), through the possession of costly structural colours (McGraw et al. 2002), or the presence of carotenoids that create bright red, orange, and yellow displays that are widely associated with signalling behaviours (Alonso-Alverez et al. 2004; McGraw et al. 2005). It has been argued that carotenoid colours are an honest signal of the condition of the individual because they provide reliable information about their health (Broggi and Senar 2009; Fargevieille et al. 2023). This is because the brightness of carotenoid-based plumage honestly reflects the condition of individuals and is therefore a widely used signal of individual health in mate selection (Fitzpatrick 1998; Hill 2000; Alonso-Alverez et al. 2004: McGraw et al. 2005; Broggi and Senar 2009; Fargevieille et al. 2023; but see McGraw et al. 2002).

Higher quality oviparous individuals may also be expected to invest more heavily in laying colourful eggs (Moreno and Osorno 2003) together with costs associated with other aspects of parental care, including nest building (Mainwaring and Hartley 2013; Cuthill et al. 2017). Calcareous reptilian eggs were thought to be white, but as dinosaurs, and birds, evolved to breed in more exposed locations, they laid more pigmented eggs for camouflage, mimicry, or individual recognition (Kilner 2006; Wiemann et al. 2018; Mainwaring et al. 2023). In extant birds, meanwhile, white eggs are typically laid by species breeding in relatively safe locations (e.g. cavities), whilst species occupying more vulnerable nest sites (e.g. open nests) often lay visually cryptic, brown eggs that are maculated (Kilner 2006; Mainwaring et al. 2015, 2023).

It has also been suggested, meanwhile, that the evolution of brightly coloured blue-green eggs is associated with female-to-male quality signalling behaviours (Moreno and Osorno 2003) or as a parasol against harmful UV rays reaching the developing embryo (Lahti and Ardia 2016). Depositing pigments into eggs is costly for female birds (Hargitai et al. 2016) and so the intensity of eggshell pigmentation positively correlates with the health of laying females (Moreno and Osorno 2003). Accordingly, many studies report that brightly coloured, and thus more pigmented, eggs are laid by higher quality females (Soler et al. 2005; Moreno et al. 2006; Siefferman et al. 2006; Martínez-de la Puente et al. 2007; Walters and Getty 2010; Hargitai et al. 2016; but see Hanley et al. 2015; Dehnhard et al. 2015). Furthermore, females laying more colourful eggs are often associated with increased levels of care by their male reproductive partners (Moreno et al. 2005; English and Montgomerie 2011; Walters et al. 2014; but see Krist and Grim 2007; Fronstin et al. 2016).

Having colourful plumages and laying colourful eggs are thus both costly because of the need for pigment deposition in generating each set of these phenotypes; in turn, we hypothesize that these costs can be borne by higher quality individuals alongside the costs of nest building. To test this positively covarying suite-of-costly-phenotype hypothesis, we used data from more than 500 species of Western Palearctic birds to test a prediction, namely that (i) bird species in which females build nests alone will be similarly colourful to males, or in other words they are less likely to be sexually plumage dichromatic. We also tested another prediction, namely that (ii) females that build nests alone will be more likely to lay colourful (pigmented) eggs to enhance their own sex-specific investment across these two early stages of reproductive costs. Finally, we used stochastic character mapping to examine the evolutionary transitions that likely occurred in the sex-specific contributions to nest building behaviours in the birds included in our study.

## Methods

### Nest building and life history data

We quantified the sex-specific nest building contributions and the colouration of parents and eggs (Table 1), from 521 species from the Birds of the Western Palearctic book series (Cramp and Simmons 1977, 1980, 1983; Cramp 1985, 1988, 1992; Cramp and Perrins 1993, 1994a, 1994b). We focused on the Western Palearctic because it was one of the few regions containing a near-complete dataset of the traits we examined at the onset of this study, and it has a variety of species that differ markedly in their sex-specific nest building contributions and plumage and egg colour (Nagy et al. 2019; Mainwaring et al. 2021; Mainwaring and Street 2021). Nest building contributions were classified as ‘male’ if males build alone, ‘female’ if females build alone, ‘both’ if both parents contribute and ‘neither’ if none of the parents contribute (i.e. seabirds laying directly onto cliff ledges).

**Table 1:** Distribution of species by categories of colouration and nest building-related variables analysed in this study

	Nest builder sex				Total
	Neither	Male	Both	Female	
Total	34	17	256	214	521
Sexual plumage dichromatism					
Yes	3	2	29	94	128
No	31	15	227	120	393
Eggshell colour					
White	26	11	168	104	309
Brown	4	5	35	26	70
Blue	4	1	53	84	142
Maculation					
Yes	19	13	170	148	350
No	15	4	85	66	170
Nest design					
Open	11	6	72	16	105
Enclosed	23	11	184	198	416

Species building less substantial nests than passerines, for example, wading birds including Eurasian oystercatchers (*Haematopus ostralegus*) and ducks including common eiders (*Somateria mollissima*), were classified into the categories above because they build nests and so exhibit sex-specific nest building contributions. Meanwhile, some species have nests that are built almost entirely by one sex, but the other sex then adds very small quantities of nest material at the end as a sexual signal, such as blue tits (*Cyanistes caeruleus*), and we have classified those nests as being built by the sex that almost exclusively built the nest (following Lifjeld et al. 2019). Finally, species in which males build multiple nests, such as the Eurasian wren (*Troglodytes troglodytes*), were classified in terms of their sex-specific building contributions to the nest in which eggs were subsequently laid, which therefore excludes their ‘display nests’ that are built to attract females and are not suitable for reproduction (following Mainwaring et al. 2021).

Sexual plumage dimorphism was classified as being present or absent, by assessing colour plates (Cramp and Simmons 1977, 1980, 1983; Cramp 1985, 1988, 1992; Cramp and Perrins 1993, 1994a, 1994b). We classified sexual plumage dimorphism in a binary manner and so species were classified as being plumage dimorphic if they had significant differences in plumage, whilst species in which females were only slightly duller than males were not classed as being dimorphic. Those species in which both sexes were similar in plumage colouration, were classified as being species in which sexual plumage dimorphism was absent. This approach relies on the assumption that for most lineages, it is the balance of natural and sexual selection that yields plumage mono- or dichromatism patterns and not the relative role of nest-building and egg-laying.

Meanwhile, we only considered the matured adult plumages of species which means that species with delayed plumage maturation (DPM) were grouped according to the ultimate, sexually mature plumage’s mono/dichromatism category. This is, however, justified as most species with DPM also display delayed reproductive investment along their life history trajectories (Hawkins et al. 2012). Thus, even though our approach is not as detailed as the approaches advocated by other studies (e.g. Dale et al. 2015), it still enables us to quantify sex-specific investment in energetically costly colourful plumage and thereby quantify the interspecific variation in the inequality of costs associated with having colourful pigmented plumage (Hernández et al. 2021). In support of this idea, Hernández et al. (2021) showed that colourful ornaments on female birds are positively associated with their male-mate preferences, clutch sizes, and their immune responses.

Clutch size ranges and the body mass of males and females were averaged per species for the analyses. Eggshell colour was determined by inspecting colour plates and defined as blue, brown, or white (Table 1). We deemed blue or brown but not beige-white as pigmented by predominantly biliverdin or protoporphyrin IX, respectively (Hanley et al. 2015), because these states of human-detectable colour variation capture not only the pigmentary composition (Verdes et al. 2015) but also the physical (Hanley et al. 2015) and avian-perceived (Wisocki et al. 2020) variability of eggshell colouration. We analysed the binary (yes/no) presence of eggshell maculation as a separate predictor in our models. Nests were classified as open when exposed as a cup or platform vs. enclosed when the latter was built inside cavities or enclosed nests comprising dome, dome and tube or burrow nests built in any location (Cramp and Simmons 1977, 1980, 1983; Cramp 1985, 1988, 1992; Cramp and Perrins 1993, 1994a, 1994b).

### Comparative analyses

We applied phylogenetically controlled generalised linear mixed models using Markov chain Monte Carlo techniques (‘MCMCglmm’ package (Hadfield 2010)) to evaluate associations between the response variable (nest building contributions, with nest builder sex coded numerically with increasing relative female contributions assumed as 1—neither, 2—male, 3—both, 4—female) and the predictor variables: sexual plumage dimorphism (present/absent: binary-coded), eggshell colour (pigmented/not: binary-coded) and maculation (spotted/not: binary-coded). We evaluated models with or without controlling for body mass and/or clutch size and/or nest design (enclosed/open: binary-coded) to examine the effect of the life history traits upon the performance of the predictors of interest. All predictors were centred and scaled prior to being entered into the model.

Ten thousand phylogenetic trees were downloaded from BirdTree (http://birdtree.org/) based on the ‘Hackett All Species’ source. The maximum clade credibility method sought a consensus tree in TreeAnnotator v1.8.3 (Rambaut and Drummond 2016), and the consensus tree was included as an inverted phylogenetic covariance matrix (Hadfield and Nakagawa 2010) in each MCMCglmm model. In all models, we used Gelman priors (Gelman et al. 2008) for the fixed effects and R=(V=1, fix=1), G=(G1=(V=1E−10, nu=−1)) priors for the residual and phylogenetic variance, respectively.

We ran most of the models for 55,000,000 iterations with 10% as burn-in and a sampling interval of 25,000 (except model 7 and 8—72,000,000 iteration with 20% as burn-in and a sampling interval of 40,000 iterations). These settings provided >>1000 posterior samples of chains for estimating the model parameters, whilst also keeping the autocorrelation level below 0.1 (Hadfield 2021).

To visualise changes in the sex of the nest builder bird and the colouration of eggshells, we mapped estimated evolutionary transitions on the phylogeny using stochastic character mapping (‘phytools’ package (Revell 2012)) of the following states: (1) non-exclusively female nest builder (neither, male, both) with white eggshell colour, (2) non-exclusively female nest builder with colourful eggshell, (3) female only nest builder with white eggshell colour, and (4) female only nest builder with colourful eggshell. We conducted a second mapping using data on nest design: (1) non-exclusively female nest builder (neither, male, both) with open nest, (2) non-exclusively female nest builder with enclosed nest, (3) female only nest builder with open nest, and (4) female only nest builder with enclosed nest. The analyses were performed in R v4.2.2 (R Core Team 2022).

## Results

**Fig. 1:**
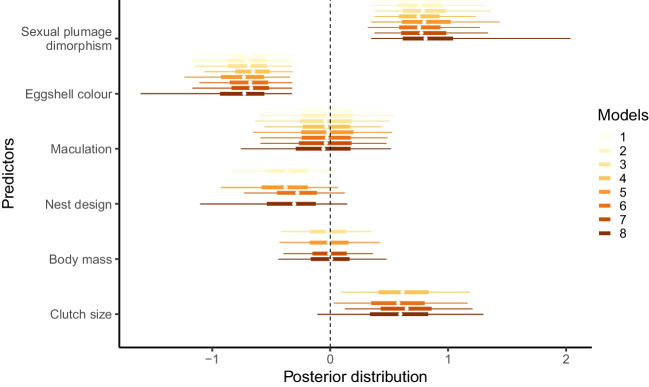
Posterior means and their 95% credible intervals of the predictors estimated in each MCMCglmm model

Sexual plumage dichromatism and eggshell colouration shows highly significant associations with sex-specific nest building contributions, even after controlling for our other predictors of body sizes, clutch sizes, or nest designs (Table 2, Fig. 1). Contrary to our first prediction, the level of female contribution to nest building is significantly higher in those species with sexual plumage dimorphism, whereas white (unpigmented) eggshells predict a significantly lower level of female contribution compared to species laying colourful and thus pigmented (blue or brown) eggs. Meanwhile, species are statistically similar in sex-specific nest building contributions whether laying maculated eggs or not. Similarly, there is no statistical difference in sex-specific nest building contributions in species building open or enclosed nests. Although species with varying levels of female contributions to nest building, with regard to species in which females build nests alone, together with their male partner or contribute nothing, have similar body sizes and clutch sizes, larger clutches suggest higher levels of female contribution to nest building (*p* < 0.1 in ¾ of the models including clutch size as predictor).

**Table 2:** Models of nest building contributions (response variable) and their predictors in phylogenetically controlled models. Posterior means (post.m) are shown for each parameter estimated in each model. Significant *p* values (*α *≤ 0.05) are marked with stars

Predictors	Model 1		Model 2		Model 3		Model 4		Model 5		Model 6		Model 7		Model 8	
	post.m	*p*	post.m	*p*	post.m	*p*	post.m	*p*	post.m	*p*	post.m	*p*	post.m	*p*	post.m	*p*
Sexual plumage dichromatism	0.781	0.002**	0.784	<0.001***	0.819	<0.001***	0.768	<0.001***	0.840	0.002**	0.772	0.001***	0.809	0.001***	0.942	0.004**
Eggshell colour	−0.695	<0.001***	−0.724	<0.001***	−0.711	0.004**	−0.668	0.004**	−0.759	0.003**	−0.694	<0.001***	−0.696	<0.001***	−0.827	0.007**
Maculation	−0.031	0.952	−0.025	0.962	−0.048	0.903	−0.042	0.902	−0.034	0.956	−0.037	0.920	−0.47	0.903	−0.077	0.885
Nest design	–	–	−0.373	0.143	–	–	–	–	−0.400	0.161	−0.292	0.245	–	–	−0.380	0.268
Body mass	–	–	–	–	−0.026	0.917	–	–	−0.005	0.960	–	–	−0.008	0.971	0.012	0.979
Clutch size	–	–	–	–	–	–	0.625	0.055	–	–	0.584	0.080	0.650	0.059	0.585	0.147

**Fig. 2:**
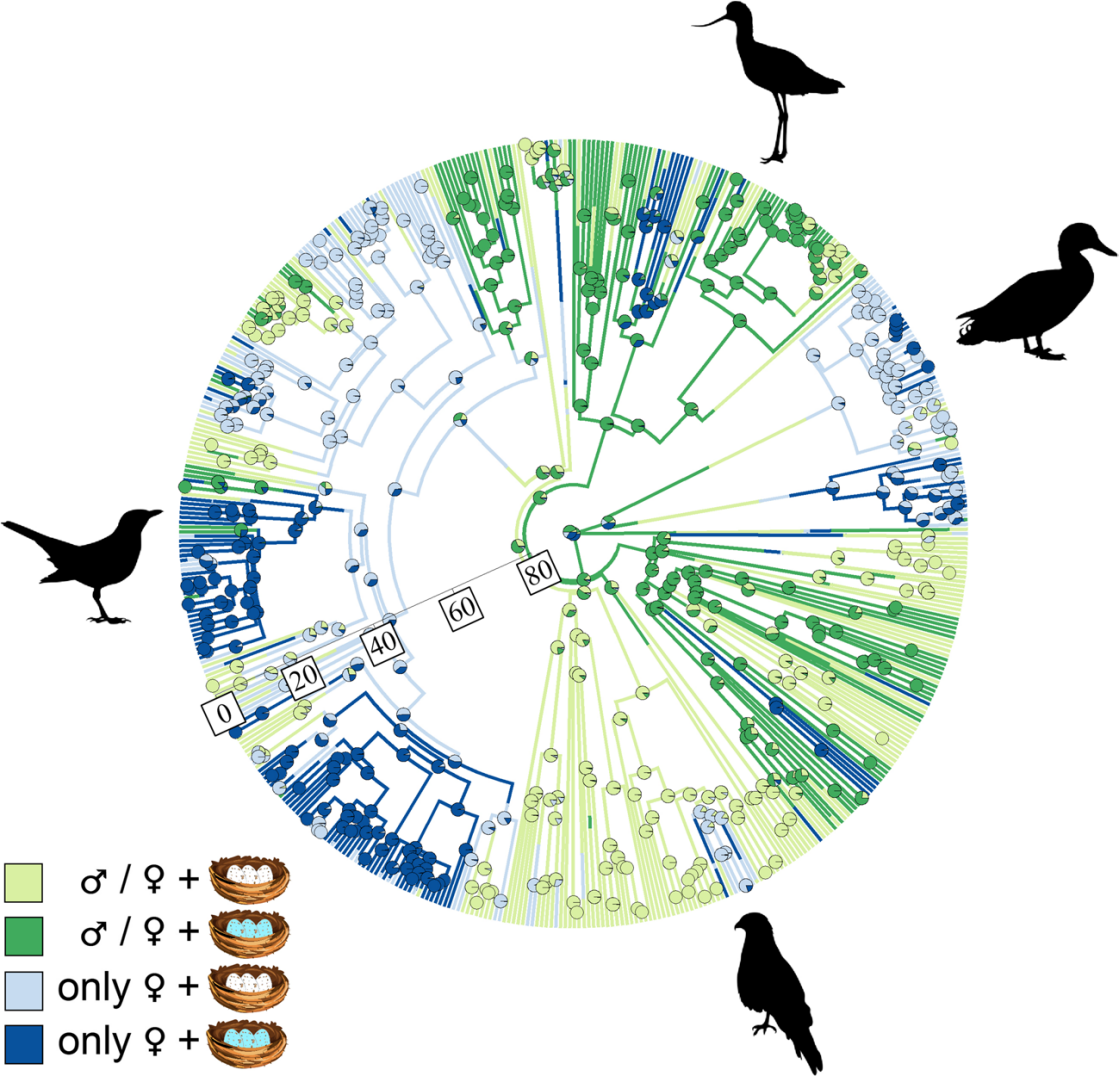
Visual representation of evolutionary changes in the relative contribution of the sex of the nest builder and the colouration of the species’ eggshells mapped on the phylogeny of 521 bird species, using the consensus of the tree sample as a backbone for character mapping. Legend from top to bottom: non-exclusively female nest builder (neither, male, both) + white eggshell, non-exclusively female nest builder + colourful eggshell, female only nest builder + white eggshell, female only nest builder + colourful eggshell

**Fig. 3:**
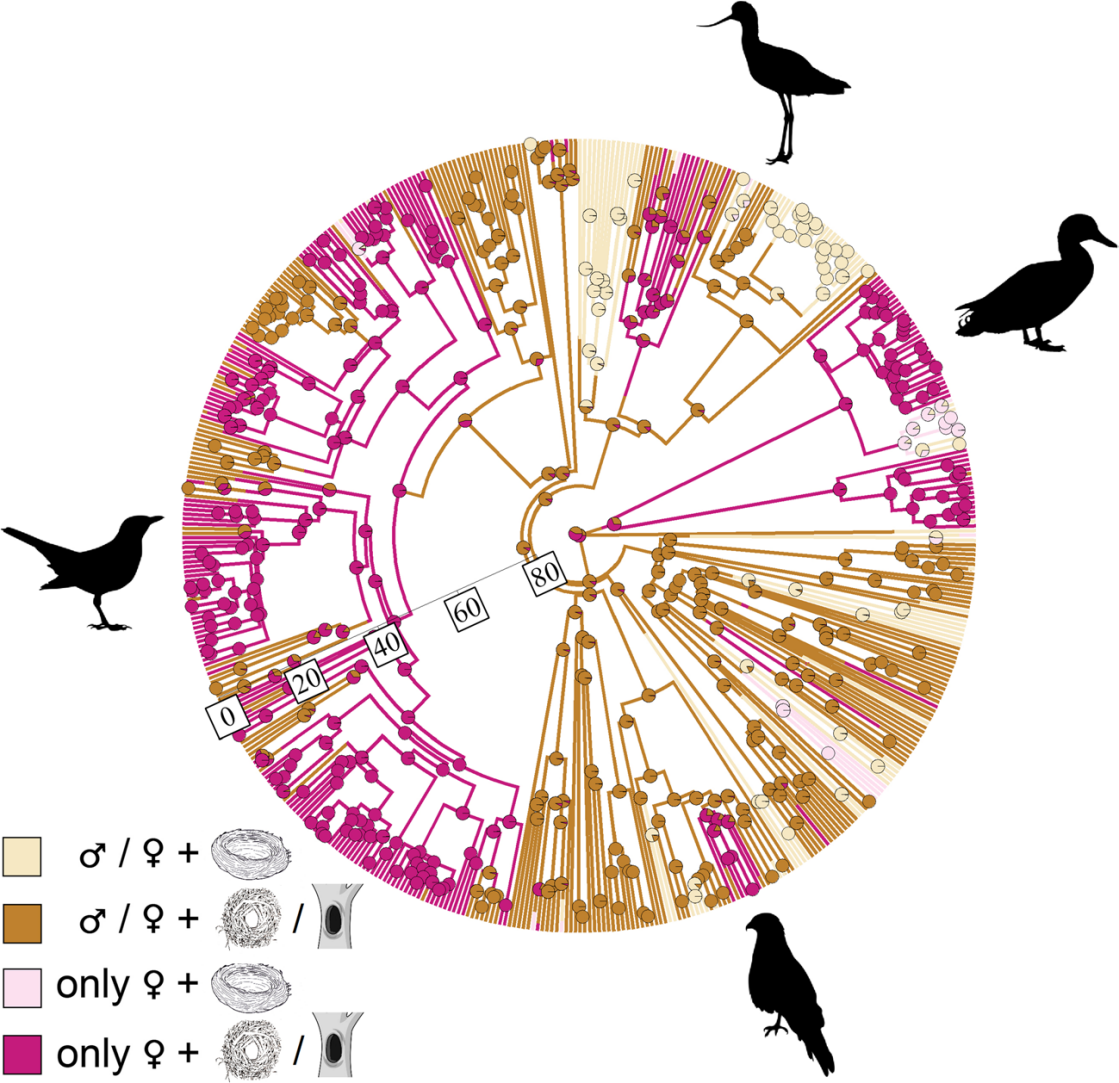
Visual representation of evolutionary changes in the relative contribution of the sex of the nest builder and the nest design mapped on the phylogeny of 521 bird species, using the consensus of the tree sample as a backbone for character mapping. Legend from top to bottom: non-exclusively female nest builder (neither, male, both) + open nest, non-exclusively female nest builder + enclosed nest, female only nest builder + open nest, female only nest builder + enclosed nest

Stochastic character mapping reveals that evolutionary transitions likely occurred from a non-exclusively female nest builder state to female only nest builder state multiple times during the evolution of the studied 521 bird species (Fig. 2). In Passeriformes, transitions from female only nest builder state with white eggshell colour to female only nest builder state with colourful eggshell were a common evolutionary pattern, but non-exclusively female nest builder state reappeared in some lineages. Evolutionary transitions from non-exclusively female nest builder state to female only nest builder state were likely to have occurred when nest design was linked to nest building contribution (Fig. 3). The female only nest builder state with open nest design probably evolved only on a few distantly related lineages. Amongst passerine species, the non-exclusively female and the female only nest builder states frequently changed, and these transitions were probably linked to more enclosed nests.

## Discussion

We show that species in which females build nests alone or together with males are more likely to have less colourful plumage in comparison to species in which females contribute less to nest building. This goes against our first prediction which was that those species in which females build nests alone would have plumage as colourful as males. Meanwhile, we also show that species in which females build nests alone or with males are more likely to lay mainly blue pigmented and, thus, more colourful (or vivid: *sensu* (English and Montgomerie 2011)) eggs, therefore providing support for our second prediction which was that species in which females invest more by building nests alone would also be investing in more colourful eggs. Finally, stochastic character mapping revealed that female-only nest building contributions repeatedly evolved during the evolution of the 521 species from the Western Palearctic that were included in this study. However, it is prudent to consider that our study taxa comprise about 5% of all the world’s bird species from a specific geographic region and that we have analysed our data using substantially pruned phylogenies. Our study will hopefully spur further studies that contain a greater portion of the world’s bird species and, thus, also take a global perspective, now that global avian nest databases are becoming increasingly available (Sheard et al. 2023; Hauber et al. 2023).

Species in which females build nests alone or together with males are more likely to have duller plumage than their male partners, whilst these patterns did not differ between species with open and enclosed nests. One possibility for these findings is that the greater investment in colourful plumage by males means they are less likely to contribute to nest building because they already invested effort in colourful plumage (Ketterson and Nolan 1994; Soler et al. 1998; Morales et al. 2011). However, the timing of the cost incurred by plumage colouration (moult) and nest building (breeding season) are often widely separated along the annual cycle of birds (e.g. Zuberogoitia et al. 2018), suggesting a lack of ecological need for such an energetic trade-off. Alternatively, it is prudent to consider that ornamentation can have a complex relationship with reproductive success (Jones et al. 2017), and some species included in this study may be expressing plumage colouration without incurring a meaningful cost (Koch et al. 2019). Further studies could therefore usefully examine these various possibilities using data from a greater portion of the world’s bird species.

An alternative explanation, meanwhile, is that colourful males may be less likely to build nests because their colourful plumage means that they are poorly camouflaged (Soler et al. 2019; Rincón-Rubio et al. 2023) and their presence may attract visually guided predators to the nest site (Mainwaring et al. 2015), who might return later to prey on the nest content when there are eggs or nestlings in it (Colombelli-Negrel and Kleindorfer 2010). In this scenario, we would have expected species with colourful males to build enclosed nests because they provide a greater shield for colourful birds from visually guided predators than open cup nests (Mainwaring et al. 2015). This, in turn, may explain why female, but not male, colouration is strongly determined by vegetative cover around nest sites that helps to provide camouflage for females (Medina et al. 2017). Although the comparative analyses of the type conducted here do not enable us to ascribe the direction of causation of the identified relationships, we nevertheless show that pre-laying reproductive investment can (negatively) covary with parental investment patterns, with species in which females invest comparatively less in colouration than their male partners investing more effort building nests.

Meanwhile, species with nests built by females alone or by males and females together were more likely to lay blue, and thus more colourful, eggs than the beige-white (unpigmented) eggs in nests where females contribute less to nest building. This suggests that females were also not balancing the costs of nest building and of having colourful plumage. It is possible that females signal their quality or condition to their male partners both via investment in laying pigmented and, thus, colourful eggs (Soler et al. 2005; Moreno et al. 2006; Siefferman et al. 2006; Martínez-de la Puente et al. 2007; Walters and Getty 2010; Hargitai et al. 2016) and via their nest building contributions. If so, then nest building contributions and egg characteristics are likely to have evolved in concert with each other, which is characterised by positively correlated evolution (Nagy et al. 2019). Further, if the nest building contributions by males enable females or even both parents to invest more effort into incubating their eggs, then the fewer visitation bouts to and from nests may mean that the eggs require less crypsis against visually guided predators and brood parasites (Kilner 2006; Morales et al. 2010).

Alternatively, the detected pattern may have also occurred if our original assumption was incorrect and pigmented eggs were not more expensive to produce than unpigmented white eggs (e.g. Hanley and Doucet 2009; Honza et al. 2011; Krištofík et al. 2013; D’Arpa et al. 2022). Such possibilities are worthy of further research because, despite the correlative, comparative relationships between eggshell colouration and reproductive investment revealed here, their causal determinants remain largely unclear. Further, stochastic character mapping revealed that evolutionary transitions towards female-only nest building occurred multiple times during the evolution of the birds included in this study. It is likely, therefore, that female-only nest building contributions are beneficial to birds (Soler et al. 2019; Mainwaring et al. 2021), although the benefits are presently unclear and are worthy of further research attention.

We have shown both that female birds’ contributions to nest building and having a colourful plumage are strongly negatively related and that females of species investing in nest building are more likely to lay colourful eggs. Our understanding of interspecific variation in sex-specific contributions to nest building is dramatically increasing (Soler et al. 1998, 2019; Lifjeld et al. 2019; Mainwaring et al. 2021, Fargevieille et al. 2023), and our study further shows that investment in colouration also covaries with sex-specific nest building contributions at the interspecific level amongst birds from the Western Palearctic region.

Nevertheless, the causal ultimate and proximate mechanisms underlying these evolutionary patterns remain unclear and so future studies could usefully examine the mechanisms underlying the comparative patterns that we have outlined here. Pertinently, it is prudent to remember that our study contained data from one region of planet earth and from about 5% of all extant bird species and so further studies that take a global approach may help confirm our findings. In this regard, studies that examine such patterns from different regions, such as the tropics, or from a global perspective may yield different results because the colouration of eggshells (Wisocki et al. 2020) and of birds (Cooney et al. 2022) varies over broad latitudinal gradients and may thus influence sex-specific nest building contributions as well. Further, intraspecific studies of species in which both parents contribute to nest building would be informative. Pertinently, quantifying sex-specific nest building contributions in relation to the experimentally altered colouration of their reproductive partners would help elucidate the proximate mechanisms underlying the comparative patterns observed in this study.

## Data Availability

Data are available from Figshare: 10.6084/m9.figshare.25268398.v1.

## References

[CR1] Alonso-Alverez C, Bertrand S, Devevey G, Prost J, Faivre B, Sorci G (2004). Increased susceptibility to oxidative stress as a proximate cost of reproduction. Ecol Lett.

[CR2] Broggi J, Senar CJ (2009). Brighter great tit parents build bigger nests. Ibis.

[CR3] Clutton-Brock TH (1991). The evolution of parental care.

[CR4] Colombelli-Négrel D, Kleindorfer S (2010). Video nest monitoring reveals male coloration-dependant nest predation and sex differences in prey size delivery in a bird under high sexual selection. J Ornithol.

[CR5] Cooney CR, He Y, Varley ZK, Nouri LO, Moody CJA, Jardine MD, Liker A, Székely T, Thomas GH (2022). Latitudinal gradients in avian colourfulness. Nat Ecol Evol.

[CR6] Cramp S (1985). The birds of the Western Palearctic 4:Terns to Woodpeckers.

[CR7] Cramp S, Perrins CM (1993) The birds of the Western Palearctic, Volume 7: Flycatchers to Shrikes. Oxford University Press, Oxford

[CR8] Cramp S, Perrins CM (1994) The birds of the Western Palearctic, Volume 8: Crows to Finches. Oxford University Press, Oxford

[CR9] Cramp S, Perrins CM (1994) The birds of the Western Palearctic, Volume 9: Buntings and New World Warblers. Oxford University Press, Oxford

[CR10] Cramp S, Simmons KEL (1977) The birds of the Western Palearctic, Volume 1: Ostrich to Ducks. Oxford University Press, Oxford

[CR11] Cramp S, Simmons KEL (1980) The birds of the Western Palearctic, Volume 2: Hawks to Bustards. Oxford University Press, Oxford

[CR12] Cramp S, Simmons KEL (1983) The birds of the Western Palearctic, Volume 3: Waders to Gulls. Oxford University Press, Oxford

[CR13] Cramp S (1988) The birds of the Western Palearctic, Volume 5: Tyrant Flycatchers to Thrushes. Oxford University Press, Oxford

[CR14] Cramp S (1992) The birds of the Western Palearctic, Volume 6: Warblers. Oxford University Press, Oxford

[CR15] Cuthill IC, Allen WL, Arbuckle K, Caspers B, Chaplin G, Hauber ME, Hill GE, Jablonski NG, Jiggins CD, Kelber A, Mappes J, Marshall J, Merrill R, Osorio D, Prum R, Roberts NW, Roulin A, Rowland HM, Sherratt TN, Skelhorn J, Speed MP, Stevens M, Stoddard MC, Stuart-Fox D, Talas L, Tibbetts E, Caro T (2017). The biology of color. Science.

[CR16] Dale J, Dey C, Delhey K, Kempenaers B, Valcu M (2015). The effects of life history and sexual selection on male and female plumage colouration. Nature.

[CR17] D'Arpa SR, Redondo I, Gómez-Llanos E, Gil D, Pérez-Rodríguez L (2022). Experimentally impaired female condition does not affect biliverdin-based egg colour. J Avian Biol.

[CR18] Dehnhard N, Pinxten R, Demongin L, Camp JV, Eens M, Poisbleau M (2015). Relationships between female quality, egg mass and eggshell blue-green colouration in southern rockhopper penguins: a test of the sexual signalling hypothesis. Polar Biol.

[CR19] English PA, Montgomerie R (2011). Robin’s egg blue: does egg color influence male parental care?. Behav Ecol Sociobiol.

[CR20] Fargevieille A, Grégoire A, Gomez D, Doutrelant C (2023). Evolution of female colours in birds: The role of female cost of reproduction and paternal care. J Evol Biol.

[CR21] Fitzpatrick S (1998) Colour schemes for birds: structural coloration and signals of quality in feathers. Ann Zool Fenn 35:67-77. https://www.jstor.org/stable/23735591

[CR22] Fronstin BR, Doucet MS, Christians KJ (2016). Haematocrit, eggshell colouration and sexual signaling in the European Starling (*Sturnus vulgaris*). BMC Ecol.

[CR23] Gelman A, Jakulin A, Pittau MG, Su YS (2008). A weakly informative default prior distribution for logistic and other regression models. Ann Appl Stat.

[CR24] Guillette LM, Healy SD (2015). Nest building, the forgotten behaviours. Curr Opin Behav Sci.

[CR25] Hadfield JD (2010). MCMC methods for multi-response generalized linear mixed models: the MCMCglmm R package. J Stat Softw.

[CR26] Hadfield JD, Nakagawa S (2010). General quantitative genetic methods for comparative biology: phylogenies, taxonomies and multi-trait models for continuous and categorical characters. J Evol Biol.

[CR27] Hadfield JD (2021) MCMCglmm course notes. Available at: https://cran.r-project.org/web/packages/MCMCglmm/vignettes/CourseNotes.pdf

[CR28] Hanley D, Doucet SM (2009). Egg coloration in ring-billed gulls (*Larus delawarensis*): a test of the sexual signaling hypothesis. Behav Ecol Sociobiol.

[CR29] Hanley D, Grim T, Cassey P, Hauber ME (2015). Not so colourful after all: eggshell pigments constrain avian eggshell colour space. Biol Lett.

[CR30] Hargitai R, Boross N, Nyiri Z, Eke Z (2016). Biliverdin-and protoporphyrin-based eggshell pigmentation in relation to antioxidant supplementation, female characteristics, and egg traits in the Canary (*Serinus canaria*). Behav Ecol Sociobiol.

[CR31] Hauber ME, Nagy J, Sheard C, Antonson ND, Street SE, Healy SD, Lala KN, Mainwaring MC (2023) Nest architecture influences host use by avian brood parasites and is shaped by coevolutionary dynamics. Proc Roy Soc B 20231734. 10.1098/rspb.2023.1734 10.1098/rspb.2023.1734 PMC10777141 38196369

[CR32] Hawkins GL, Hill GE, Mercadante A (2012). Delayed plumage maturation and delayed reproductive investment in birds. Biol Rev.

[CR33] Hernández A, Martínez-Gómez M, Beamonte-Barrientos R, Montoya B (2021). Colourful traits in female birds relate to individual condition, reproductive performance and male-mate preferences: a meta-analytic approach. Biol. Lett..

[CR34] Hill GE (2000). Energetic constraints on expression of carotenoid-based plumage coloration. J Avian Biol.

[CR35] Honza M, Požgayová M, Procházka P, Cherry IM (2011). Blue-green eggshell coloration is not a sexually selected signal of female quality in an open-nesting polygynous passerine. Naturwissenschaften.

[CR36] Jones JA, Tisdale AC, Bakermans MH, Larkin JL, Smalling CG, Siefferman L (2017). Multiple plumage ornaments as signals of intrasexual communication in Golden-Winged Warblers. Ethology.

[CR37] Ketterson ED, Nolan V (1994). Male parental behaviour in birds. Annu Rev Ecol Evol Syst.

[CR38] Kilner RM (2006). The evolution of egg color and patterning in birds. Biol Rev.

[CR39] Koch RE, Staley M, Kavazis AN, Hasselquist D, Toomey MB, Hill GE (2019). Testing the resource trade-off hypothesis for carotenoid-based signal honesty using genetic variants of the domestic canary. J Exp Biol.

[CR40] Krist M, Grim T (2007). Are blue eggs a sexually selected signal of female collared flycatchers? A cross-fostering experiment. Behav Ecol Sociobiol.

[CR41] Krištofík J, Darolová A, Griggio M, Majtán J, Okuliarová M, Zeman M, Zídková L, Hoi H (2013). Does egg colouration signal female and egg quality in reed warbler (*Acrocephalus scirpaceus*)?. Ethol Ecol Evol.

[CR42] Lahti DC, Ardia DR (2016). Shedding light on bird egg color: pigment as parasol and the dark car effect. Am Nat.

[CR43] Lifjeld JT, Gohli J, Albrecht T, Garcia-Del-Rey E, Johannessen LE, Kleven O, Marki PZ, Omotoriogun TC, Rowe M, Johnsen A (2019). Evolution of female promiscuity in Passerides songbirds. BMC Evol Biol.

[CR44] Mainwaring MC, Hartley IR (2013). The energetic costs of nest building in birds. Avian Biol Res.

[CR45] Mainwaring MC, Street SE (2021). Conformity to Bergmann’s rule in birds depends on nest design and migration. Ecol Evol.

[CR46] Mainwaring MC, Hartley IR, Lambrechts MM, Deeming DC (2014). The design and function of birds’ nests. Ecol Evol.

[CR47] Mainwaring MC, Nagy J, Hauber ME (2021). Sex-specific contributions to nest building in birds. Behav Ecol.

[CR48] Mainwaring MC, Medina I, Tobalske BW, Hartley IR, Varricchio DJ, Hauber ME (2023). Evolution of nest site use and nest architecture in birds and their non-avian ancestors. Phil Trans Roy Soc B.

[CR49] Mainwaring MC, Reynolds SJ, Weidinger K (2015) The influence of predation on the location and design of nests. In D. C. Deeming & S. J. Reynolds (Eds.), Nests, eggs, and incubation: new ideas about avian reproduction 50-64. Oxford University Press, Oxford

[CR50] Martínez-de la Puente J, Merinon S, Moreno J, Tomás G, Morales J, Lobato E, García-Fraile S, Martínez J (2007). Are eggshell spottiness and colour indicators of health and condition in blue tits *Cyanistes caeruleus*?. J Avian Biol.

[CR51] McGraw KJ, Mackillop EA, Dale J, Hauber ME (2002). Different colors reveal different information: how nutritional stress affects the expression of melanin- and structurally based ornamental plumage. J Exp Biol.

[CR52] McGraw KJ, Hill EG, Parker SR (2005). The physiological costs of being colourful: nutritional control of carotenoid utilization in the American Goldfinch (*Carduelis tristis*). Anim Behav.

[CR53] Medina I, Delhey K, Peters A, Cain KE, Hall ML, Mulder RA, Langmore NE (2017). Habitat structure is linked to the evolution of plumage colour in female, but not male, fairy-wrens. BMC Evol Biol.

[CR54] Morales J, Torres R, Velando A (2010). Parental conflict and blue egg coloration in a seabird. Naturwissenschaften.

[CR55] Morales J, Velando A, Torres R (2011). Biliverdin-based egg colouration is enhanced by carotenoid supplementation. Behav Ecol Sociobiol.

[CR56] Moreno J, Osorno LJ (2003). Avian egg colour and sexual selection: does eggshell pigmentation reflect female condition and genetic quality?. Ecol Lett.

[CR57] Moreno J, Morales J, Lobato E, Merino S, Tomás G, Martínez-de la Puente J (2005). Evidence for the signaling function of egg color in the Pied Flycatcher *Ficedula hypleuca*. Behav Ecol.

[CR58] Moreno J, Lobato E, Morales J, Merino S, Tomás G, Martínez-de la Puente J, Sanz JJ, Mateo R, Soler JJ (2006). Experimental evidence that egg color indicates female condition at laying in a songbird. Behav Ecol.

[CR59] Nagy J, Hauber ME, Hartley IR, Mainwaring MC (2019). Correlated evolution of nest and egg characteristics in birds. Anim Behav.

[CR60] R Core Team (2022) R: a language and environment for statistical computing. R Foundation for Statistical Computing, Vienna, Austria. URL: http://www.R-project.org/

[CR61] Rambaut A, Drummond AJ (2016) TreeAnnotator v1.8.3. University of Edinburgh, Edinburgh, UK. http://beast.bio.ed.ac.uk/treeannotator/

[CR62] Revell LJ (2012) phytools: an R package for phylogenetic comparative biology (and other things). Methods Ecol Evol 3:217-223. http://www.respond2articles.com/MEE/

[CR63] Rincón-Rubio VA, Székely T, Liker A, Gonzalez-Voyer A (2023). Carotenoid-dependent plumage coloration is associated with reduced male care in passerine birds. Behav Ecol.

[CR64] Sheard C, Street SE, Healy SD, Troisi CA, Clark AD, Yovcheva A, Trébaol A, Vanadzina K, Lala KN (2023). Nest traits for the world’s birds. Glob Ecol Biogeog.

[CR65] Siefferman L, Navara KJ, Hill GE (2006). Egg coloration is correlated with female condition in Eastern Bluebirds (*Sialia sialis*). Behav Ecol Sociobiol.

[CR66] Soler JJ, Møller AP, Soler M (1998). Nest building, sexual selection and parental investment. Evol Ecol.

[CR67] Soler JJ, Moreno J, Avilés JM, Møller AP (2005). Blue and green egg-color intensity is associated with parental effort and mating system in passerines: support for the sexual selection hypothesis. Evolution.

[CR68] Soler JJ, Morales J, Cuervo JJ, Moreno J (2019). Conspicuousness of passerine females is associated with the nest-building behaviours of males. Biol J Linn Soc.

[CR69] Verdes A, Cho W, Hossain M, Brennan PLR, Hanley D, Grim T, Hauber ME, Holford M (2015). Nature’s palette: characterization of shared pigments in colorful avian and mollusk shells. PLoS ONE.

[CR70] Walters LA, Getty T (2010). Are brighter eggs better? Egg color and parental investment by house wrens. J Field Ornithol.

[CR71] Walters LA, Olszewski N, Sobol K (2014). Male house wrens provide more parental provisioning to nests with a brighter artificial egg. Wilson J Ornithol.

[CR72] Wiemann J, Yang TR, Norell MA (2018). Dinosaur egg colour had a single evolutionary origin. Nature.

[CR73] Wisocki PA, Kennelly P, Rivera IR, Cassey P, Burkey ML, Hanley D (2020). The global distribution of avian eggshell colours suggest a thermoregulatory benefit of darker pigmentation. Nat Ecol Evol.

[CR74] Zuberogoitia I, Zabala J, Martínez JE (2018). Moult in birds of prey: a review of current knowledge and future challenges for research. Ardeola.

